# Detailed molecular cytogenetic characterisation of the myeloid cell line U937 reveals the fate of homologous chromosomes and shows that centromere capture is a feature of genome instability

**DOI:** 10.1186/s13039-020-00517-y

**Published:** 2020-12-14

**Authors:** Ruth N. MacKinnon, Joanne Peverall, Lynda J. Campbell, Meaghan Wall

**Affiliations:** 1 Victorian Cancer Cytogenetics Service, St Vincent’s Hospital, PO Box 2900, Fitzroy, Melbourne, 3065 Australia; 2 Department of Medicine, St Vincent’s Hospital, University of Melbourne, Parkville, Australia; 3 PathWest Department of Diagnostic Genomics, PathWest Laboratory Medicine, QEII Medical Centre, Nedlands, Australia; 4 Victorian Clinical Genetics Services, Parkville, Melbourne, Australia; 5 Murdoch Children’s Research Institute, Parkville, Melbourne, Australia

**Keywords:** U937, Cell lines, Centromere capture, Genome evolution, Acute myeloid leukemia, Molecular karyotype, Dicentric chromosomes

## Abstract

**Background:**

The U937 cell line is widely employed as a research tool. It has a complex karyotype. A *PICALM*-*MLLT10* fusion gene formed by the recurrent t(10;11) translocation is present, and the myeloid common deleted region at 20q12 has been lost from its near-triploid karyotype. We carried out a detailed investigation of U937 genome reorganisation including the chromosome 20 rearrangements and other complex rearrangements.

**Results:**

SNP array, G-banding and Multicolour FISH identified chromosome segments resulting from unbalanced and balanced rearrangements. The organisation of the abnormal chromosomes containing these segments was then reconstructed with the strategic use of targeted metaphase FISH. This provided more accurate karyotype information for the evolving karyotype. Rearrangements involving the homologues of a chromosome pair could be differentiated in most instances. Centromere capture was demonstrated in an abnormal chromosome containing parts of chromosomes 16 and 20 which were stabilised by joining to a short section of chromosome containing an 11 centromere. This adds to the growing number of examples of centromere capture, which to date have a high incidence in complex karyotypes where the centromeres of the rearranged chromosomes are identified. There were two normal copies of one chromosome 20 homologue, and complex rearrangement of the other homologue including loss of the 20q12 common deleted region. This confirmed the previously reported loss of heterozygosity of this region in U937, and defined the rearrangements giving rise to this loss.

**Conclusions:**

Centromere capture, stabilising chromosomes pieced together from multiple segments, may be a common feature of complex karyotypes. However, it has only recently been recognised, as this requires deliberate identification of the centromeres of abnormal chromosomes. The approach presented here is invaluable for studying complex reorganised genomes such as those produced by chromothripsis, and provides a more complete picture than can be obtained by microarray, karyotyping or FISH studies alone. One major advantage of SNP arrays for this process is that the two homologues can usually be distinguished when there is more than one rearrangement of a chromosome pair. Tracking the fate of each homologue and of highly repetitive DNA regions such as centromeres helps build a picture of genome evolution. Centromere- and telomere-containing elements are important to deducing chromosome structure. This study confirms and highlights ongoing evolution in cultured cell lines.

## Background

MacGrogan et al. [1] published a study of several acute myeloid leukaemia (AML) cell lines with loss of heterozygosity (LOH) at 20q12, to delineate the common deleted region found in myeloid malignancies [1]. We have carried out a detailed characterisation of the genomes of two of these cell lines, HEL [2] and U937, using a molecular cytogenomics approach. As well as confirming the del(20)(q12) reported in the karyotypes, these studies demonstrate the combined use of different molecular methods to characterise chromosome rearrangements in detail.

U937 was established from the pleural fluid of a patient with “diffuse histiocytic lymphoma”. Despite the label of “lymphoma”, the patient’s malignant cells contained eosinophilic granules and resembled blast cells of the monocytic lineage, expressing myeloid markers. The cell line is commonly used to study myeloid differentiation [3–5].

Shipley et al. [6] published karyotypes for three separate sublines including one from the laboratory of origin [3]. More recently, several karyotypes have been published using a combination of chromosomal CGH (comparative genomic hybridisation, the precursor of the higher resolution technique of array CGH) [7] and fluorescence in situ hybridisation (FISH) [8–11].

There is considerable variation between these published karyotypes. The abnormalities which are not common to all sublines may have arisen in vitro. However, as we will discuss, there are also some differences in interpretation of the karyotype due to the techniques used for analysis.

The abnormal gene formed by fusion of the *MLLT10* (*AF10*) and *PICALM* (*CALM*) genes was discovered in this cell line [12] and presumably arose in vivo. The recurrent translocation, t(10;11)(p12;q14), which created this fusion, is present in all published karyotypes. This translocation occurs in diverse haematological malignancies including acute lymphoblastic leukaemia and acute myeloid leukaemia [12, 13].

In this report we combine data from G-banding, FISH (fluorescence in situ hybridisation), M-FISH and M-BAND (multicolour-FISH and -banding respectively) and SNP (single nucleotide polymorphism) array to describe the abnormal chromosomes in detail. This study corrects a previous interpretation [1] of the chromosome 20 abnormality in U937.

The U937 genome has been sequenced and the data are available online from the Cancer Cell Line Encyclopedia [14].The present study complements the sequencing data as it characterises the abnormal chromosomes with a focus on chromosome structure and evolution, particularly with reference to the centromeres, details which are not learned from the sequence data. The karyotype presented here corrects or gives greater detail than the previously published karyotypes.

## Results

**Fig. 1:**
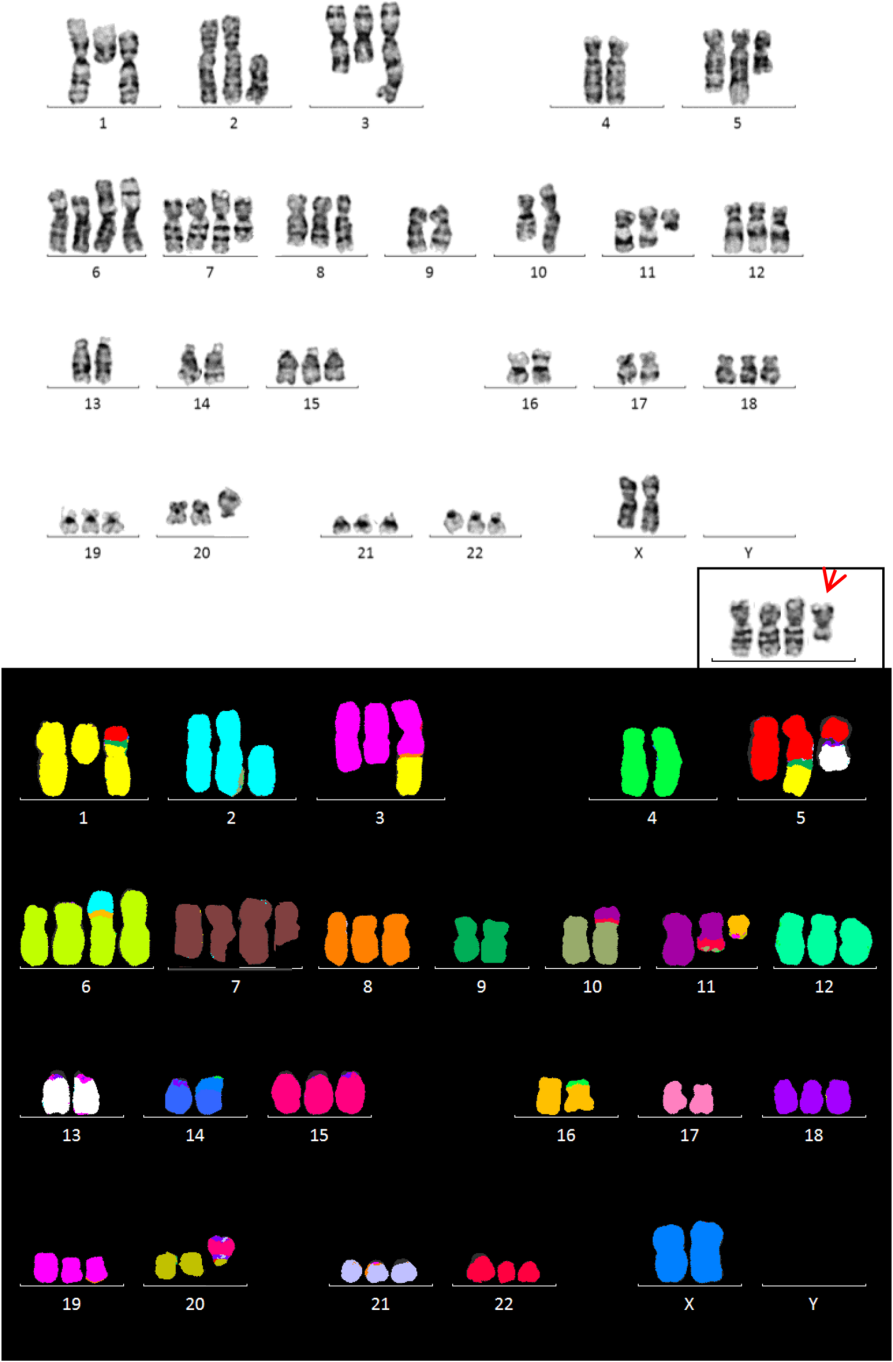
G-banded (top) and M-FISH images of the U937 karyotype (bottom) (second cell line). The chromosomes are positioned in the karyotype according to their known or assumed centromere identity. The inset in the G-banded image shows the der(7)t(6;7) (arrows)

**Fig. 2:**
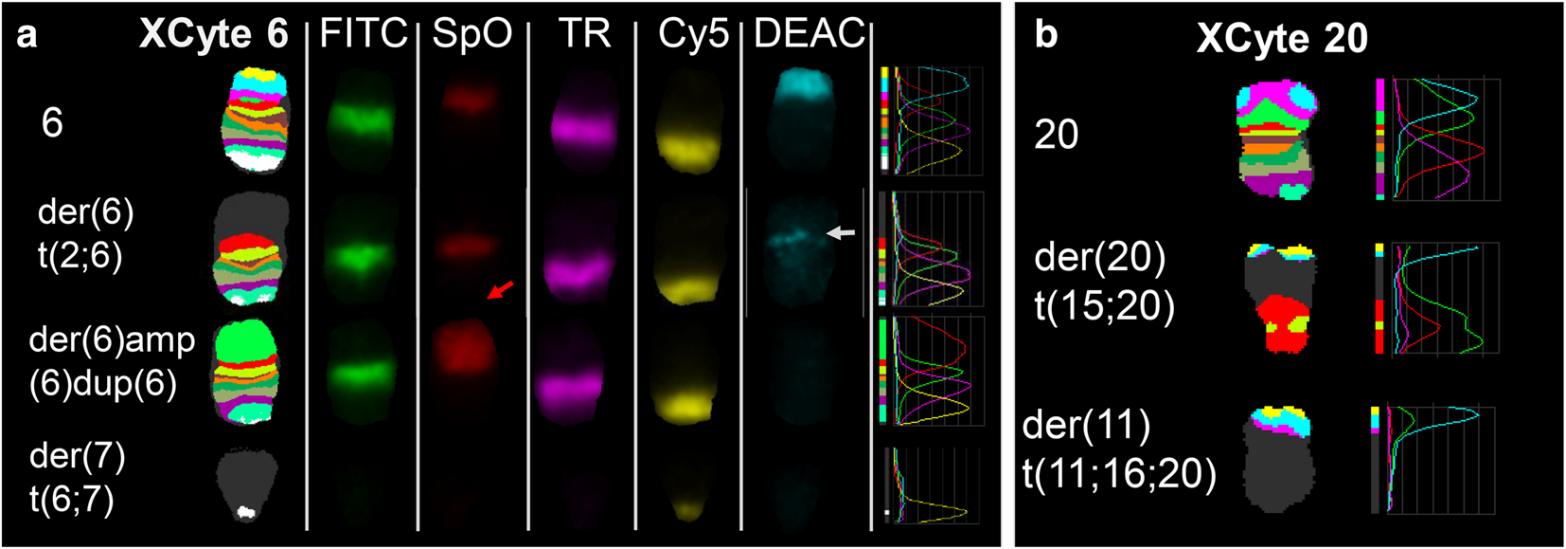
M-BAND images for chromosomes 6 and 20 for U937 chromosomes. **a** XCyte 6 images. Left to right, M-BAND 6 image from a metaphase with a der(6)del(6)dup(6), (top three chromosomes), and a der(7) from a different metaphase (bottom chromosome). Left to right, five single colour galleries, fluorescence intensity profiles. The closed white arrow points to a thin band in the aqua channel (brightened to make it visible in the image) of the der(6)t(2;6), showing that this chromosome has a more distal breakpoint (i.e. closer to the telomere of the short arm) than the der(6)del(6)dup(6), which does not have any aqua signal. The expanded red signal in the der(6)del(6p)dup(6) reveals amplification of 6p12-> 6p21 material (red arrow). **b** XCyte 20 images. Left to right, M-BAND image, fluorescence intensity profiles showing the presence of chromosome 20 material in the der(20)t(15;20) and the der(11)t(11;16;20)

**Fig. 3:**
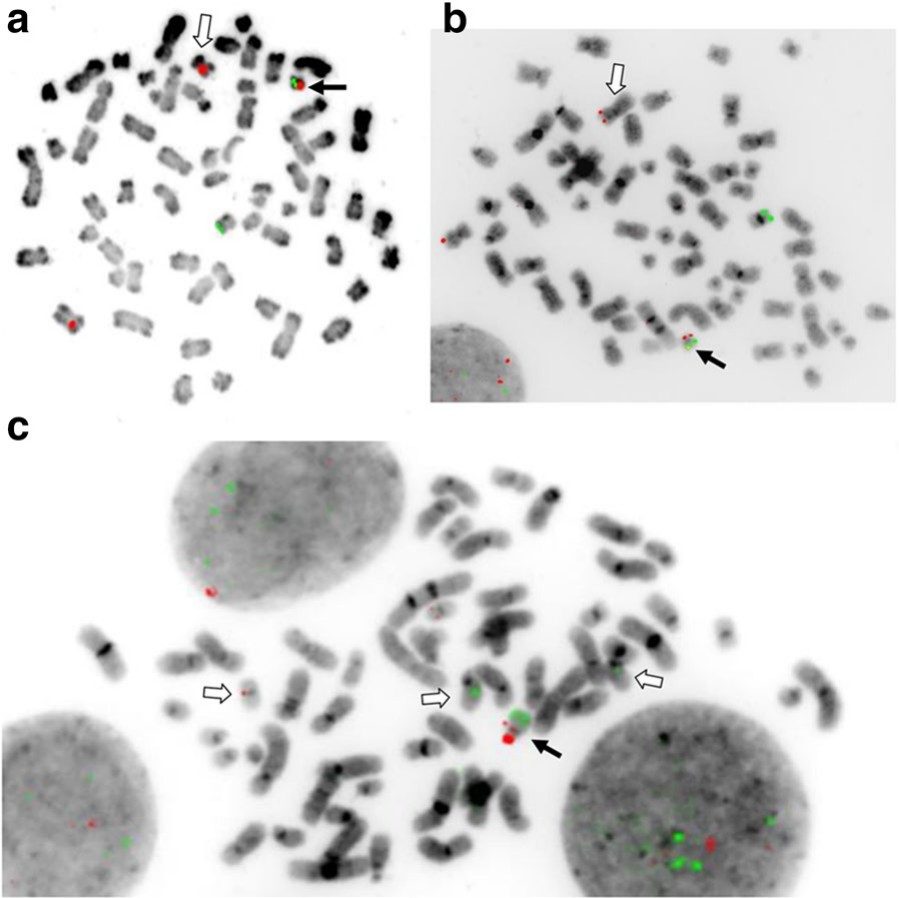
Representative metaphase FISH images clarifying the der(11)t(11;16;20) and der(20)(t(15;20) in U937. **a** A chromosome 11 centromere probe (red) and an *MYH11* (16p13) probe (green) shows that the 11 centromere is in the der(11)t(11;16;20) (closed arrow). The open arrow indicates the der(11)t(10;11). A normal 11 and 16 are also present (labelled with red and green respectively). (Texas Red signal from the *CBFB* part of the CBFβ/MYH11 probe was not visualised with the filters used for this image.) **b** The BAC probe RP11-754N12 (11q24.3, representing *ETS1*) (red) and an *MYH11* (16p13) probe (green) show that the extra copy of 11q24.3 material is on the der(11)t(11;16;20) (closed arrow). Open arrow, der(10)t(10;11). A normal 11 and 16 are also present (labelled with red and green respectively). **c** The 20p11.21 BAC probe RP11-500O11 (red) and 15q21.2 BAC probe RP11-607G03 (green) show the patterns of amplification and duplication respectively of these regions on the der(20)t(15;20) (solid arrow). Normal chromosomes 15 and 20 are indicated by open arrows

Representative G-banded and M-FISH karyotypes and M-BAND images are presented in Figs. 1 and 2. Targeted FISH results are shown in Tables 1 and 2 and examples are illustrated in Fig. 3.

**Table 1:** Results of FISH for derivative chromosomes containing chromosome 20 segments

1a			Abnormal chromosome	
Band	Chr position (GRCh38)	Marker/clone	der(11)t(11;16;20)	der(20)t(15;20)
11 centromere		*D11Z1*	+	–
11q23.3	11:118436490-118525221	*KMT2A (MLL)*	–	–
11q24.3	11:128311087-128485203	RP11-754N12 (*ETS1)*	+	–
11q24.3	11:128694094-128812000	*FLI1*	+	–
16p13.11	16:15703135-15857030	*MYH11*	+	–
15q14	15:37089671-37297139	RP11-597G23	+^a^	
15q21.2	15:51495000-51629079	RP11-607G03	++^a^	
15q22.2	15:60041031-60218374	RP11-366L09	(+)^b^	
15q25.1	15:80970069-81137763	RP11-775C24	++^a^	
20p11.21	20:22409751-22462800	RP11-500O11	–	++++^b^
20p11.21	20:22756464-22940284	RP11-755M18	–	+++^b^
20p11.21	20:23530430-23671508	RP11-218C14	–	++^b^
	20:24130923-24332313	RP11-717H21	–	+
	20:24568986-24763965	RP11-580L12	–	+
	20:25124608-25306738	RP11-156D15	–	+
	20:25306022-25435715	RP11-384D7	–	+
20p11.1	20:25925848-26084581	RP11-269F15	–	+
20 centromere		*D20Z1*	–	+
20q11.21	20:31245645-31409837	RP11-602P9	–	+
	20:31435456-31585732	RP11-802B20	–	+
	20:31483476-31684958	RP11-363M16	(+)	+
	20:31592650-31760766	RP11-702M08	+	+
	20:31705215-31874074	RP11-243J16	+	+
	20:31902727-32053101	RP11-71I02	(+)	+
	20:31935170-32131327	RP11-620H13	(+)	+
	20:32022592-32204778	RP11-483M19	–	+
	20:32385910-32580055	RP11-724J12	–	+
	20:33132629-33305883	RP11-49G10	–	+
20q11.22	20:33458641-33609412	RP11-120F10	–	–
	20:33747044-33935043	RP11-541L2	–	–
	20:34110945-34261427	RP11-642P13	–	–
20q12	20:42201817-42202000	D20S108	–	–

Each + represents a signal intensity equivalent to one copy on the normal chromosome. (+) represents reduced intensity signal. Positions for each BAC are those given in ensembl.org archives converted to GRCh38 co-ordinates. The order of chromosome 15 and 20 elements in the der(20)t(15;20) is derived from FISH and is described in the karyotype (Table 3)

^a^Very faint signal—the breakpoint is at distal end of this clone

^b^One copy of each is present in the long arm of the der(20) and the additional copies are in the short arm

**Table 2:** Results of FISH for other derivative chromosomes

Chromosome band	Chromosome position (GRCh38)	Marker/clone	Chromosome			
			psu dic(3;1)	del(1)(q11)	der(5)t(1;5)	der(5)t(5;13)
1 centromere			+ (inactive^a^)	+		
5q11.1	5:50139424-50285096	RP11-185I4				(+)
5q11.1	5:50768911-50961846	RP11-317O24			+	+ (15/20)/- (5/20) metaphases
+ (69/100)/- (31/100) interphases						
5q11.2	5:57029637-57212477	RP11-101B14			+	+ (15/20)/- (5/20) metaphases
5q11.2	5:58287078-58423150	RP11-313I12			+	+

Chromosome band	Chromosome position (GRCh38)	Marker/clone	Chromosome		
			Cytogenetically normal 7 (one copy)	Normal 7 (other 2 or 3 copies)	der(7)t(6;7)(q27;q21.12) or del(7)(q22.1q34)
7p15	7:24505360-24515863	RP11-343P21	++	+	+

Chromosome band	Chromosome position (GRCh38)	Marker/clone	Chromosome	
			Cytogenetically normal 8 (one copy)	Normal 8 (other 2 copies)
8p	8:475607-658638	RP11-800L13	++	+

Each + represents a signal intensity equivalent to one copy on the normal chromosome; ++ represents a double-strength signal; (+) represents reduced intensity signal

^a^As inferred by the absence of a centromeric constriction

**Fig. 4:**
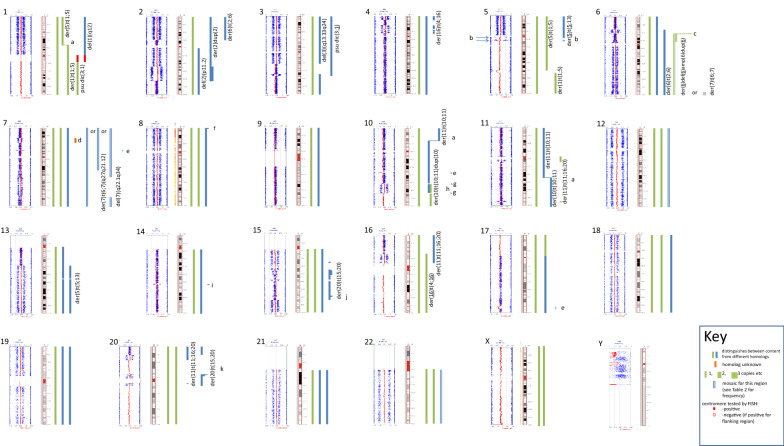
SNP array images and interpretation for each chromosome. SNP array results are arranged by chromosome. For each chromosome the output from the Karyostudio software is displayed against the ideogram. Vertical bars aligned to the right of the ideograms define each segment of the chromosome and its location in a normal or abnormal chromosome. The abnormal chromosomes containing each region are identified when possible. Chromosomes derived from the same homologue are represented in bars of the same colour when this could be determined. N.B. inheritance from the same parent cannot be inferred for different chromosomes represented by the same coloured vertical bar. When breakpoints were adjacent to the centromere, presence or absence of the centromere (chromosome 1) is shown by a solid (present) or hollow bar as shown in the key, if tested. **a** Horizontal line, balanced translocation, **b** the regions represented by B allele frequencies of 0:0.25:0.75:1 (arrowed) were deleted from the der(5)t(5;13) in approximately 30% of cells, **c** B allele frequency and approximately 50% mosaicism for this chromosome suggest approximately ten copies of the most highly amplified section (see Results), **d** This duplicated region was on a chromosome 7 without the 7q deletion (homologue unknown), **e** Deleted chromosome not identified, **f** gain of a short sub-telomeric 8p section was on a cytogenetically normal chromosome 8, **g** one copy only, presumed on the normal 10, **h** to explain the AAB/ABB pattern here, we have assumed that the der(10) is most likely to contain both homologues, as conversion of the 10qter segment suggests that the duplication was derived by an unbalanced translocation between the two homologues. **i** Location of this segment unknown, **j** additions and deletions of 15q assumed most likely to be on the der(20) reflecting its heritage involving breakage and rejoining events, rather than the normal 15 homologue represented in blue, **k** inverted repeat of the 20p amplified region. For a high resolution version of Fig. 4, see Additional file 1

The SNP array results are presented in Fig. 4 together with interpretations made by correlation with banding and FISH results. We used the log R ratio and B allele frequency information provided by SNP array, as well as information provided by FISH, to establish copy number, assign the breakpoints of most unbalanced rearrangements, and distinguish between the two homologues of chromosomes with abnormalities. Chromosome positions reported here refer to build GRCh38.

SNP array data for this cell line showed that most copy number aberrations were present in all cells. Three major clones were identified by G-banded karyotyping and these mosaic chromosomes were confirmed by B allele frequency data. B allele frequencies were used to estimate copy number when there was uncertainty, as they are more sensitive than the log R ratio.

The karyotype compiled from seventy G-banded karyotypes and interpreted with the aid of M-FISH, locus-specific FISH and SNP array data is presented in Table 3.

**Table 3:** The U937 karyotype

62<2n> ,
X, +X,−Y,
del(1)(q12),
+der(1)t(1;5)(p22;**q31.1**),
del(2)(p11.2),
+der(2)dup(2)(**q24.1q33.1**)del(2)(**q33.1**),
del(3)(**q13.33q24**),
+psu dic(3;1)(**q25.1**;p11.1),
der(5)t(1;5)(p22;**q23.3**), +der(5)t(5;13)(**q11.2**;**q14.11**)del(5)(**q11.2q11.2**)^1^,
+der(6)t(2;6)(**p13.2**;**p22.1**),
+ 7, +dup(7)(p15.3p15.1),
+ 8,
der(10)t(10;11)(p12.31;q14.2)t(10;10)(**q23.33q25.2**)^2^,
der(11)t(10;11)(p12.31;q14.2),
+der(11)(**16pter**->**16p11.2**::**11p11.12**->**11q12**::**11q24.12**->**11q24.2**::**20q11.21**->**20q11.21**::**20p12.3**->**20pter**)^3^,
+ 12,
+ 15,
der(16)t(4;16)(**p13**;**p12.2**)del(4)(**p14p14**)del(4)(**p15.1p15.1**)del(4)(**p15.31p16.1**),
+ 18,
+ 19,
der(20)(20pter->20p12.2::15q14->15q25.3::20p11.22->20q11.21::20p11.21->20p11.21:)^4^
+ 21,
+ 22 [21] /
63,idem, +der(6)del(6)(**p21.31**)amp(6)(**p21.31**)dup(6)(**p21.31p12.2),** del(7)(**q22.1q34**)[37] /
60,idem, der(7)t(6;7)(**q27;q21.12**), − 12, − 22[13]

The karyotype was compiled from seventy G-banded karyotypes and interpreted with the aid of M-FISH, locus-specific FISH and SNP array data. Bold text indicates breakpoints that were determined from SNP array data. Balanced translocations were detected by M-FISH and refined by M-BAND and/or G-banding but no information on their breakpoints was provided by SNP array data. The breakpoints of the reciprocal t(10;11) are known by the location of the involved genes. A p → q orientation is assumed when no information is available

^1^The complex SNP array pattern on proximal 5q (Fig. 4) was resolved by FISH (Table 2). There were two different deletions of 5q11.2 from the der(5)t(5;13). The smaller deletion occurred in 70% of cells and the larger overlapping deletion in 30% of cells (see Fig. 4 and Table 2). It was not determined which clones these derivatives belong to

^2^Submicroscopic deletions of chromosome 10 are noted: homozygous deletion at 10q22.2, del(10)(q22.2q22.2) (chromosome unknown), del(10)(q23.33q23.33) and del(10)(q25.2q25.2). The latter two deletions flank the duplication and therefore we assume they are on the der(11)t(10;11)

^3^The position given for the 11q24.1-> 11q24.2 segment in the der(11)t(11;16;20) is based on FISH data (Fig. 3b)

^4^This derivative has submicroscopic deletions of 15q14->15q21.1, 15q22.2->15q22.2, submicroscopic duplications of 15q21.1->15q21.3, 15q25.1->15q25.2, and duplication and amplification of a subsection of 20p11.21 in the short arm of the derivative (see Figs. 3, 4, Table 1)

There were additional submicroscopic abnormalities identified by SNP array (identified by SNP array Fig. 4; some of these were located by FISH—see Tables 1 and 2) and not included in the karyotype, including: duplication of the subtelomeric segment 8p23.3->pter on an apparently normal chromosome 8, deletion of 13q23.31 from an apparently normal chromosome 13 and the der(5)t(5;13), gain of 14q24.1 material in an unknown chromosome, gain and loss of 15 and 20 material in the der(20)t(15;20) (see footnote to Table 3) and deletion of 17q25.2->q25.3.

In this male-derived cell line the X chromosome is duplicated and there was no Y chromosome. We have reported the organisation of the chromosome 2 abnormalities previously [15].

The karyotype of our U937 subline was hyperdiploid/hypotriploid, consistent with other published karyotypes. Although the karyotype suggests a past triploidisation event, it is written based on gain or loss of chromosomes from diploidy, to allow comparison with previously published karyotypes written from diploidy [6, 8–11]. An extra copy of most chromosomes was represented. Figure 4 shows the content of the abnormal chromosomes in either blue or green, representing each of the two homologues, information which was deduced from the corresponding B allele frequency pattern. Most chromosome reorganisation events involved the duplicated homologue, showing that these rearrangements occurred after triploidisation. Exceptions are chromosome 1 (deletion of the minor homologue), chromosomes 10 and 11 (the *PICALM*-*MLLT10* fusion event, see below), possibly chromosome 20, and chromosomes 5, 6 and 16 of which both homologues were rearranged. In addition, the only abnormal chromosome duplicated at the triploidisation event was a chromosome 13 with a submicroscopic deletion (Fig. 4).

The balanced t(10;11), producing *MLLT10*-*PICALM* fusion, was present, and the derivative chromosome 10 had undergone a duplication event, also reported by Lee et al. [9] and shown in more detail here. This duplication appears to have arisen via a non-homologous recombination event with the other chromosome 10 homologue (the presumed third copy of chromosome 10), as this derivative is comprised of the other homologue below the duplicated region (see Fig. 4). The exchange involved deletion of the regions immediately flanking the duplicated segment.

There was trisomy of chromosomes 8, 18, 19 and 21 and the cell line was mosaic for trisomy 12 and trisomy 22, the der(6)del(6)amp(6)dup(6), del(7), der(7)t(6;7) and a deletion of the der(5)t(5;13) (see below).

Rearrangements involving chromosomes 5, 6, 7, 8, 11, 13, 15, 16 and 20 were resolved by FISH and SNP array as detailed below and in Tables 1 and 2 and Fig. 4.

### Chromosome 5

The proximal long arm of chromosome 5 showed a complex SNP array pattern. This was interpreted using FISH with BACs for the regions marked with arrows in Fig. 4, revealing that these regions were present in the der(5)t(5;13) in 70% of cells (Table 2), and therefore deleted from this chromosome in the other 30% of cells (Fig. 4).

### Chromosome 6

There were two apparently normal copies of chromosome 6 (one of each homologue, represented by blue and green vertical bars in Fig. 4) and three abnormal derivatives involving both homologues.

M-BAND places the chromosome 6 breakpoint on the der(6)t(2;6) distal to the breakpoint on the der(6)del(6)amp(6)dup(6) (hereafter referred to as a der(6)del(6)dup(6)) (Fig. 2). The amplification and duplication of 6p on the der(6)del(6)dup(6) accounts for the broad SpectrumOrange signal in the M-BAND image (red arrow in Fig. 4). Amplification or gain of proximal 6p or 6p21 in U937 has been reported by others [9–11].

The der(6)del(6)dup(6) was only present in about 50% of cells and there were more copies of a 6q subtelomeric segment than the rest of 6q. The short subtelomeric 6q segment was identified by M-BAND in a proportion of the cells without the der(6)del(6)dup(6). We reasoned that this telomere was used to cap another mosaic chromosome in cells without the der(6)del(6)dup(6). The prime candidate was the chromosome 7 with the larger deletion, as it was mosaic and a telomere was not accounted for. The chromosome 6 M-BAND pattern confirmed that this subtelomeric segment was on a chromosome matching this chromosome’s morphology. This chromosome, hereafter named a der(7)t(6;7), was present in a clone without the der(6)del(6)dup(6), but was derived from the other chromosome 6 homologue (the homologue involved in the der(6)t(2;6)—see below).

The chromosome 6 copy number and B allele frequency patterns in the SNP array plot were matched with M-BAND and karyotype data to define the homologues in these abnormal chromosomes as follows:

- 6pter->6p22: two copies and completely heterozygous (allele pattern AB), i.e. in the normal chromosomes 6 only;
- 6p22->6p21.3: three copies, (allele patterns AAB and ABB), the extra copy being in the der(6)t(2;6);
- 6p21.3 amplified region: the pattern is consistent with an eight allele pattern of AABBBBBB and AAAAAABB; the two copies of the minor homologue are accounted for by a normal chromosome 6 and the der(6)t(2;6); one copy of the major homologue is accounted for by the other normal chromosome 6, and four copies by the amplified chromosome; as the der(6)del(6)dup(6) was present in approximately 50% of cells it has about eight copies of this amplified region;
- 6p21.3->6p12.3 four copies, AABB; one homologue is accounted for by a normal chromosome 6 and the der(6)t(2;6); and the other homologue by the other normal chromosome 6 and two copies in the duplicated region on the der(6)del(6)dup(6) (present in about 50% of cells);
- 6p12.3->6q26, three-and-a-half copies, i.e. 1.5A:2B and 2A:1.5B (one copy in the der(6)del(6)dup(6) in half of the cells);
- 6q26->6qter gain compared to 6p12.3->6q26; this segment is present in the der(6)del(6)dup(6) and the der(7)t(6;7) (see Fig. 4); the B allele frequency pattern trends towards heterozygosity here compared to the rest of 6q, therefore this segment is derived from the most frequent homologue at this point, i.e. the homologue involved in the der(2)t(2;6) and represented in blue (Fig. 4).

### Chromosome 7

There were four copies of chromosome 7, and in some cells one of two different rearrangements had occurred, independently producing overlapping partial loss of 7q. These two variants were reported as deletions in a previous FISH study [15] but the current study resolves one as an unbalanced translocation with chromosome 6 forming a der(7)t(6;7), respectively, as described above (see Chromosome 6 section; Fig. 4).

FISH with the BAC RP11-343P21 (7p15) together with the Metasystems XL del(7)(q22q31) probe that detects 7q deletion confirmed that the extra copy of 7p15 material was located on one of the apparently normal chromosomes 7 (Fig. 1 and Table 2).

### Chromosome 11

In addition to a normal chromosome 11 and the t(10;11) there was a third copy of a 6 Mb region of chromosome 11 which spanned the 11 centromere (Fig. 4), which suggested that a third 11 centromere was present at an unknown location, presumably derived from a chromosome 11 that had been duplicated at triploidisation. FISH confirmed that there was an 11 centromere on a short submetacentric chromosome (Fig. 3b). A chromosome identified by M-FISH as consisting of 20p and 16p material matched this chromosome morphologically, and since SNP array showed that neither the chromosome 16 nor chromosome 20 content spanned its respective centromere (Fig. 4), this was a strong candidate for the chromosome with the third 11 centromere. This was confirmed by FISH with the Vysis 11 centromere probe (CEP11 (D11Z1) SpectrumOrange) together with the Aquarius CBFβ/MYH11 translocation probe used as a marker for this abnormal chromosome (Fig. 3a). This chromosome should therefore be described as a der(11)t(11;16;20). A shorter, 1.6–2 Mb, section from 11q24.2->11q24.3 [The proximal boundary was between base pair positions 11:127,168,221 and 11:127,348,488 (GRCh38) and the distal boundary between 11:129,066,363 and 11:129,199,648 (GRCh38)] containing the *ETS1* and *FLI1* oncogenes, was also present on this abnormal chromosome (Figs. 3b, 4). As these two sections of chromosome 11 were separated by the 10;11 translocation, we can conclude that they were derived from the normal “green” homologue (Fig. 4) rather than the homologue involved in the t(10;11). Presumably they are a remnant of the normal chromosome 11 that was duplicated during triploidisation.

### Chromosome 17

According to the Cancer Cell Line Encyclopedia [14], *TP53* in U937 has the recurrent C>T mutation at hsa17:7675052 (GRCh38) (NM_001126112.2(TP53):c.559+1G>A). Our SNP array data show that although there were two copies of chromosome 17, there was complete loss of heterozygosity for most of the chromosome 17 short arm, including *TP53*. Therefore, this allele is homozygous with two copies in a pleudotriploid karyotype. One copy of chromosome 17 had a cryptic 17q25 deletion.

### Chromosome 20

There were two normal copies of one chromosome 20 homologue. The third copy of chromosome 20, representing the other homologue, had undergone complex rearrangements resulting in loss of the 20q12 region encompassing the myeloid common deleted region [16], and net loss from triploidy of this region. This result confirmed LOH of this region as reported by MacGrogan et al. [1]. One to four copies of parts of 20pter->20q11.21 were present in two abnormal chromosomes: the der(11)t(11;16;20) described above and a der(20) containing elements of chromosomes 15 and 20 including the 20 centromere (Figs. 2, 4, Tables 1 and 2). The third copy of the 20q11.21 common retained region [17] was distributed between these two abnormal chromosomes (Table 1, Fig. 2, 3c, 4). There was no simple deleted chromosome 20.

## Discussion

Our study has provided a more accurate and detailed description of the chromosome abnormalities in U937. Our method included SNP array to identify rearranged segments, metaphase FISH to localise these segments and further characterise abnormal chromosomes, and B allele frequencies to distinguish between rearrangements of the two different homologues. B allele frequency data from SNP array analysis also allows a comparison of the number of copies of each homologue of a chromosome pair, and is sometimes useful for estimating copy number.

For most chromosomes, the contribution of each of the two homologues could be differentiated using B allele frequency data. Importantly, when there was more than one abnormality of a chromosome, B allele frequency data often allowed us to determine whether these involved the same or different homologues (as demonstrated in Fig. 4). For example, two different chromosome 16 rearrangements that could have potentially involved the same chromosome 16 were shown to instead involve not only two different chromosomes 16 but also the two different homologues (Fig. 4). Another case in point is the der(10)t(10;11): the portion of chromosome 10 distal to the duplication is comprised of material from the other 10 homologue, i.e. there was gene conversion of this region (see Fig. 4). This duplication appears to have arisen via an unbalanced translocation between the der(10)t(10;11) and one copy of the duplicated normal chromosome 10 presumed to have been formed at triploidisation (“green” homologue), with loss of material at the breakpoints (see Results and Fig. 4).

### Centromere capture

We described centromere capture events for the first time, in complex unbalanced karyotypes, where acentric segments from one or more chromosomes were preserved by joining to a centromere from a different chromosome [2, 18], and this concept was also later reported by Garsed et al. [19]. A centromere is necessary for stable inheritance and survival of a chromosome formed by the repair of broken chromosome segments [18]. Neocentromeres are functional centromeres created de novo by chromatin modification, and appear to perform a similar function, i.e. rescue of chromosomes that have no centromere [20]. Marker chromosomes with neocentromeres have been described in various sarcoma subtypes [20, 21].

Telomere capture is a similar concept that has been described in cancers and is well accepted [22, 23]. In this cell line we were able to match a short subtelomeric segment from 6q with a deleted chromosome 7 that had no apparent 7q telomere. On SNP array the subtelomeric segment acted as a proxy for the telomere.

We have previously described four centromere capture events: in two unbalanced translocations in the cell line HEL [18] and in two anachromosomes (chromosomes produced by chromothripsis) in a case of AML [2]. The present study identifies a further example of centromere capture: acentric segments from chromosomes 16 and 20 were identified in an abnormal chromosome, which had a centromere from chromosome 11.

These five examples of centromere capture were identified in highly rearranged genomes which we studied with a focus on identifying ambiguous centromeres. As this approach to chromosome characterisation is uncommon, centromere capture may be a significant feature of complex karyotypes. Centromere capture may provide a mechanism for the rescue of broken or shattered chromosome material, providing a selective advantage to the cancer cell [2]. If it provides a mechanism for preservation of oncogenes after chromothripsis or other chromosome breakage events, it may be much more common than these few cases indicate, since the identity of centromeres is not usually studied [2, 19, 21]. When there are multiple breakage and repair events occurring together, for example during chromothripsis [24], the surviving chromosome segments may simply be those that have joined to a segment containing a centromere. Deleted segments would therefore be those that do not re-join to segments containing a centromere and an appropriate telomere complement [18].

### U937 heritage

U937 was first described in 1976 [3], but the karyotypes of U937 sublines held in different laboratories varied considerably from one another by the time they were karyotyped in 1988 [6]. Shipley et al. [6] analysed G-banded chromosomes of three sublines held at different laboratories, U937-1, U937-2, and U937-3. The t(10;11), del(3q) and der(16)t(4;16) were common to all three sublines and were also present in our specimen, and there were several unresolved markers in each subline.

Several later publications [8–11, 25] refined the karyotype using different combinations of chromosomal CGH and FISH. The abnormal chromosomes described in all of these publications also included a der(1) and a der(5) from a translocation between chromosomes 1 and 5 (described as unbalanced in our study with evidence from microarray data and in another publication using chromosomal CGH [8], but balanced in other studies), a del(2p), a psu dic(3;1) (otherwise described as a dic(1;3) [8] or der(3)t(1;3) 9–11]), the der(6) with 6p amplification and a der(6)t(2;6). With the exception of the del(2p) these abnormalities were all described in the U937-1 karyotype of Shipley et al. [6, 9], and none were described in U937-2 or U937-3. This suggests that the sublines characterised in these later publications and the present study, sourced from both the ATCC (American Type Culture Collection) and the DSMZ (German Collection of Microorganisms and Cell Cultures) [8–11] were closer to each other and to U937-1 than to U937-2, or to U937-3 which was obtained from the laboratory that established U937 [3, 6].

There were several other abnormalities that were described in some studies only. Although some of these differences can be explained by different approaches to analysis, as described below, the detail of some suggests that they are true differences. For example, Cottier et al. [8] described a secondary translocation of the der(6)t(2;6) with chromosome 18; several authors reported a der(6)t(6;12) [8] or dic(6;12) [9, 11] which was not present in our subline. The del(1q), a fourth copy of chromosome 7 and a third copy of chromosome 22 (mosaic) were unique to our study. There was some additional mosaicism in our subline. This highlights the continuing evolution of cell line genomes in vitro. As a consequence, sublines held in other laboratories may differ in detail from the one described here.

Lee et al. [9] identified duplication of the 2q31->2q33 region in a der(2)dup(2)(q31q33)t(2;6)(q33;q21) by reverse chromosome painting (characterising abnormal chromosomes by labelling and hybridising them to normal metaphase spreads), a duplication that we also identified in our subline. However, they reported a subsequent unbalanced translocation with chromosome 6, a rearrangement not present in our specimen. (Both specimens shared a different 2;6 translocation.)

### Refined and redefined abnormalities

Comparing the written and photographed karyotypes of the different publications is challenging, and it is not always clear which differences can be attributed to evolution and which to karyotyping inaccuracy. Like the fable of the Blind Men and the Elephant [26], abnormalities of the genome can be described and understood in different ways depending on the tools and the resolution obtained. The U937 genome has been the subject of several characterisations by G-banding, M-FISH, CGH, and/or SNP array [6, 8–11], and sequencing data are available [14]. Here we highlight some similarities and differences between our and published U937 karyotypes that can be explained by different approaches to analysis.

Descriptions of an abnormal chromosome characterised by different assays can be unrecognisable as the same chromosome. This is illustrated by the following two abnormal chromosomes.

One abnormal chromosome whose description has varied depending on the techniques used was a chromosome that was first described by G-banding as a “del(17p)” by Shipley et al. [6]. We identified a der(20)t(15;20), which had a 20 centromere together with chromosome 15 and 20 material, by M-FISH, M-BAND and 20 centromere FISH. Identifying only the chromosome 15 material using a whole chromosome 15 paint, Lee et al. [9] identified a “der(15)” (i.e. a derivative chromosome with a 15 centromere). The inversion and deletion breakpoints that they gave this chromosome using CGH (comparative genomic hybridisation, a FISH technique using labelled cell line DNA pre-annealed to normal DNA to probe normal chromosomes, to identify copy number changes [7]) data are in good agreement with our SNP array data (see Fig. 4), but they did not identify the chromosome 20 content in this abnormal chromosome. Using both chromosome 15 and chromosome 20 paints and a 20 centromere probe, Matteucci et al. [11] identified it as a der(20) (i.e. having a 20 centromere) with elements of chromosomes 15 and 20 but without any detail on breakpoints.

Matteucci et al. [11] identified the chromosome 20 content of the der(11)t(11;16;20) using a chromosome 20 paint, and described it as a der(20). However Lee et al. [9], using a chromosome 16 paint, identified it as a del(16q). Stefford et al. [10] identified both the chromosome 16 and chromosome 20 components of this chromosome with M-FISH, which identifies components of all chromosomes. The combination of SNP array, M-FISH and M-BAND enabled a cohesive and more accurate description of this chromosome. M-BAND data showed that the der(16)t(4;16) had the higher of two chromosome 16 breakpoints (Figs. 1, 4). Using B allele frequency and breakpoint data from SNP array we could distinguish between the two 16p breakpoints on different chromosomes in the U937 genome and ascertain that the corresponding der(4)t(4;16) and der(11)t(11;16;20) were derived from different chromosome 16 homologues, the der(4)t(4;16) being derived from the duplicated homologue. We also showed that the der(11)t(11;16;20) contained an 11 centromere by FISH, based on clues from SNP array data and confirmed by FISH (Fig. 3a).

Using various techniques including M-FISH but not FISH for the 20 centromere, Cottier et al. [8] reported that their DSMZ-derived U937 subline had three normal chromosomes 20, and they did not identify any chromosome matching our subline’s chromosome 20-containing abnormal chromosomes (the der(20)t(15;20) and the der(11)t(11;16;20))—these might be absent in the DSMZ subline. Shipley et al. [25] described three chromosomes that were positive for an 11 centromere: the normal 11, an isochromosome, i(11) and an E-group chromosome. The “isochromosome” matches the der(11)t(10;11) morphologically, and their E-group chromosome fits the description of our der(11)t(11;16;20), which is positive for *ETS1*. However, they did not identify *ETS1* on this chromosome, nor did they identify it on the der(10)t(10;11), neither of which was known to contain chromosome 11 material at the time [27]. Gene localisation by tritiated in situ hybridisation is relatively insensitive and chromosome identification is difficult (personal observation), so that positive signals on an unexpected chromosome could easily have been missed (the authors discussed this possibility [6]).

Of interest, MacGrogan et al. [1] reported loss of heterozygosity at the 20q12 common deleted region (CDR) (they do not specify whether they used specimen from the ATCC or DSMZ) but three copies of the YAC 834H3 region, leading them to conclude that there had been loss of the CDR followed by reduplication from the other homologue. We cannot find mapping information for 834H3 but suggest either that it is not in the region that was lost, or, less plausibly, that reduplication occurred in the subline they tested but not in ours.

### Independent 7q deletion producing no net loss of 7q

Trisomy 7 and/or deletion of 7q has been reported in most other U937 specimens [8, 9, 11], but our specimen alone had a fourth copy of chromosome 7. Partial loss of 7q occurred twice, independently: once as a del(7q) in the largest clone, which had a der(6)del(6)dup(6), and independently in a different, minor clone that did not have the der(6)del(6)dup(6), by unbalanced translocation of chromosome 7 with a copy of the other chromosome 6 homologue (Fig. 4, Table 2). The occurrence of 7q deletion twice independently is consistent with 7q deletion conferring a selective advantage to the cell. Deletion of 7q is a recognised recurrent myeloid deletion, but in this cell line loss of 7q from one of four copies of chromosome 7 produced partial loss of heterozygosity but no net deletion from the pseudotriploid background. This apparent paradox may be worth further investigation.

### Analysis of complex genome reorganisation

Large high-throughput studies of cancer cell lines are producing publicly available expression, copy number and sequence data, and are a valuable resource for understanding cell line biology [28–30]. Standard sequencing technologies cannot yet analyse regions of highly repetitive DNA [31]. Nor do cytogenomic microarrays give information on chromosome organisation. Metaphase FISH is a single cell analysis tool which can help fill in some of these gaps. More recently, optical mapping [32] and nanopore sequencing [33–37] are making the description of highly complex karyotypes more comprehensive, and these will allow the exploration of chromosome rearrangements with greater resolution, including long read sequencing across centromeres. One advantage of our approach is that the distribution of the homologues can readily be interpreted. It is also more accessible at the present time, and targeted portions of the genome can be examined as needed.

A viable chromosome has two telomeres and at least one centromere. To help build a picture of the abnormal chromosomes, ideally an abnormal chromosome will include two subtelomeric segments identified by SNP array data, which account for the two telomeres. However, as the telomeres are highly repetitive and are not themselves represented on the array, the subtelomere cannot always be used as a proxy for the telomere. For example we assume the der(2)dup(2) has a telomere, even though the 2q subtelomere has been lost (Fig. 4); and we found the subtelomere of 8p to be duplicated on an apparently normal chromosome 8 in our U937. There were several chromosomes without obvious telomeres (i.e. without two subtelomeric segments), including the del(1), the der(2)dup(2), the del(2p), the der(6)del(6)dup(6), and the der(20)t(15;20). Centromere FISH performed on metaphase chromosomes can identify centromeres. If one of the SNP array segments in a chromosome does not contain a centromere or join to a chromosome segment with a centromere, centromere capture or a neocentromere should be suspected.

In 2013, two comprehensive studies of the complex and widely used HeLa genome [38, 39] were published. In one of these studies, Adey et al. [38] used haplotypes of isolated chromosomes, allele ratios and mate-pair sequencing to distinguish between the different chromosome homologues in the abnormal chromosomes and determine the probable structure of marker chromosomes, although the centromere content of the marker chromosomes was not identified. This haplotype information importantly allowed the authors to conclude that *MYC* was cis-activated by the inserted HPV18 (human papilloma virus) DNA in this cervical cancer cell line. As in our study, this is an example where distinguishing between alterations on the two alternative homologues can provide information on how the genome changes arose.

The present study is valuable as a demonstration of the analysis of complex rearrangements, and also the evolution and main features of the U937 genome. However, it cannot be a definitive picture of the U937 genome due its continuing evolution, as demonstrated by the variation between different sublines and the examples of mosaicism in this subline. Landry et al. [39] predicted that in future, cell line genomes will be routinely characterised so that changes can be identified and studies of cellular processes can be related to the actual genome rather than the reference genome. Studying how genomes in cell lines, cancers and mouse models of cancer are remodelled, should help us understand the processes of karyotype evolution. We advocate the use of a variety of complementary methods to characterise abnormalities and identify the processes occurring during karyotype evolution.

## Conclusions

We have demonstrated how a combination of SNP array and metaphase FISH techniques can produce more detail of the chromosome reorganisation of complex unbalanced karyotypes than either technique on its own.

This approach has allowed us to identify the fate of each chromosome homologue, and to show that tracking the fate of highly repetitive DNA regions such as centromeres can help us build a picture of genome evolution and the importance of centromere- and telomere-containing elements. Our data complement the sequencing and DNA array-based data that are publicly available.

Using our approach, chromosome segments and breakpoints can be assigned to different abnormal chromosomes, and some evolutionary steps can be inferred. The fate of the two homologues of a chromosome can often be determined. FISH can also be used to confirm predictions and clear up uncertainties, for example, the location of centromeres and short isolated segments.

We have identified one or more examples of centromere capture every time we have used this approach to study a cancer genome which has undergone complex rearrangement. In U937 we identified a chromosome formed with segments derived from chromosome 16 and 20 without their respective centromeres, which had been preserved by cryptic joining to a 6 Mb segment containing an 11 centromere. As centromere capture is not recognised by any common analytical procedure, we suggest that this method of preserving acentric fragments that would otherwise be lost is common in genomes with highly rearranged chromosomes, for example, in cancers with genome instability, and may be selected for when it preserves an oncogene on a broken chromosome.

## Methods

U937 cells were cultured in RPMI containing 10% FCS, glutamine, penicillin and streptomycin in air containing 5% CO_2_, and harvested to produce metaphase chromosomes using standard procedures [40]. Chromosomes were G-banded using Leishman’s stain according to standard techniques [41]. The karyotype was described according to ISCN [42].

BACs were selected on the basis of their map positions in the Ensembl database (http:/GRCh37.ensembl.org/index.html) and labelled with SpectrumOrange and/or SpectrumGreen (Abbott Molecular, Downers Grove, IL) to generate locus-specific FISH probes mapping to the regions of interest. All BACs were checked for chromosomal location before use, and were hybridised at a final concentration of 10–15 ng/µL.

Other locus-specific probes included *D20S108* from the common deleted region at 20q12 [16] (Vysis LSI D20S108 (20q12) SpectrumOrange, Abbott Molecular), the Aquarius CBFβ/MYH11 Translocation, Dual Fusion Probe (Cytocell, Cambridge), the Vysis LSI MLL dual colour, break apart probe (11q23), an 11 centromere probe (CEP11 (D11Z1) SpectrumOrange, Abbott Molecular), centromere-specific probes for chromosome 20 (Vysis CEP20 (D20Z1) SpectrumOrange, Abbott Molecular; and Poseidon SE20 D20Z1 (aqua), Kreatech, Amsterdam), chromosomes 1, 5 and 19 (Poseidon SE1/5/19 (Green) (Kreatech), CEP8 (D8Z2) SpectrumOrange (Abbott Molecular) and probes for the 7 centromere and 7q myeloid common deleted region (Vysis LSI D7S486 (7q31) SpectrumOrange/CEP7 SpectrumGreen, Abbott Molecular; and XL del(7)(q22q31), Metasystems). Combinations of two or three probes labelled with contrasting fluorochromes were hybridised to metaphase chromosomes using the Vysis co-denaturation protocol with co-denaturation at 72 °C for 2 min. When combining Poseidon probes with Vysis or BAC probes, the amount of Poseidon probe used (not diluted with the buffer provided) was one-tenth to one-fifth of the final volume, the recommended Poseidon pre-treatment protocol was used, and probes and chromosomes were co-denatured at 73 °C for 3 min and washed according to the Vysis protocol [17].

M-FISH and M-BAND were carried out using XCyte probes (Metasystems, Altlussheim, Germany) according to the manufacturer’s instructions.

FISH was analysed using a Zeiss Axioplan 2 fluorescence microscope, and captured with a Metasystems Isis capturing and analysis station. Signal level for single locus probes was estimated by comparing the signal on the abnormal chromosome to the signal on the normal chromosome. The number and relative intensity of signals per cell was estimated and averaged over at least ten metaphases.

SNP array was carried out on the Illumina CytoSNP 12 platform, with DNA extracted from cultured cells using a DNeasy Cell and Tissue kit (Qiagen, Germantown, MD) in the log phase of growth.

G-band, M-BAND and M-FISH images and karyotypes were compared with published images and karyotypes to determine abnormalities in common between U937 sublines in different laboratories. Chromosome composition and breakpoints were determined using the G-band and M-FISH images, SNP array data, and M-BAND and single locus FISH data when available. SNP array data have been deposited at the Genome Expression Omnibus (GEO) with accession number GSE41964.

In most instances the precise breakpoints of copy number aberration and unbalanced translocations could be determined from SNP array data showing changes in copy number and B allele frequencies. M-BAND and single locus FISH helped locate short chromosome segments below the resolution of M-FISH.

## Supplementary Information

**Additional file 1: ** High resolution version of Fig. 4.

## Availability of data and materials

SNP array data are available at the Genome Expression Omnibus (GEO) with accession Number GSE41964 https://www.ncbi.nlm.nih.gov/geo/query/acc.cgi?acc=GSE41964.
