# Supplementary figures and images for: Detailed molecular cytogenetic characterisation of the myeloid cell line U937 reveals the fate of homologous chromosomes and shows that centromere capture is a feature of genome instability
Source: Mol Cytogenet. 2020 Dec 14;13:50. doi: 10.1186/s13039-020-00517-y (PMC7737353; doi:10.1186/s13039-020-00517-y)

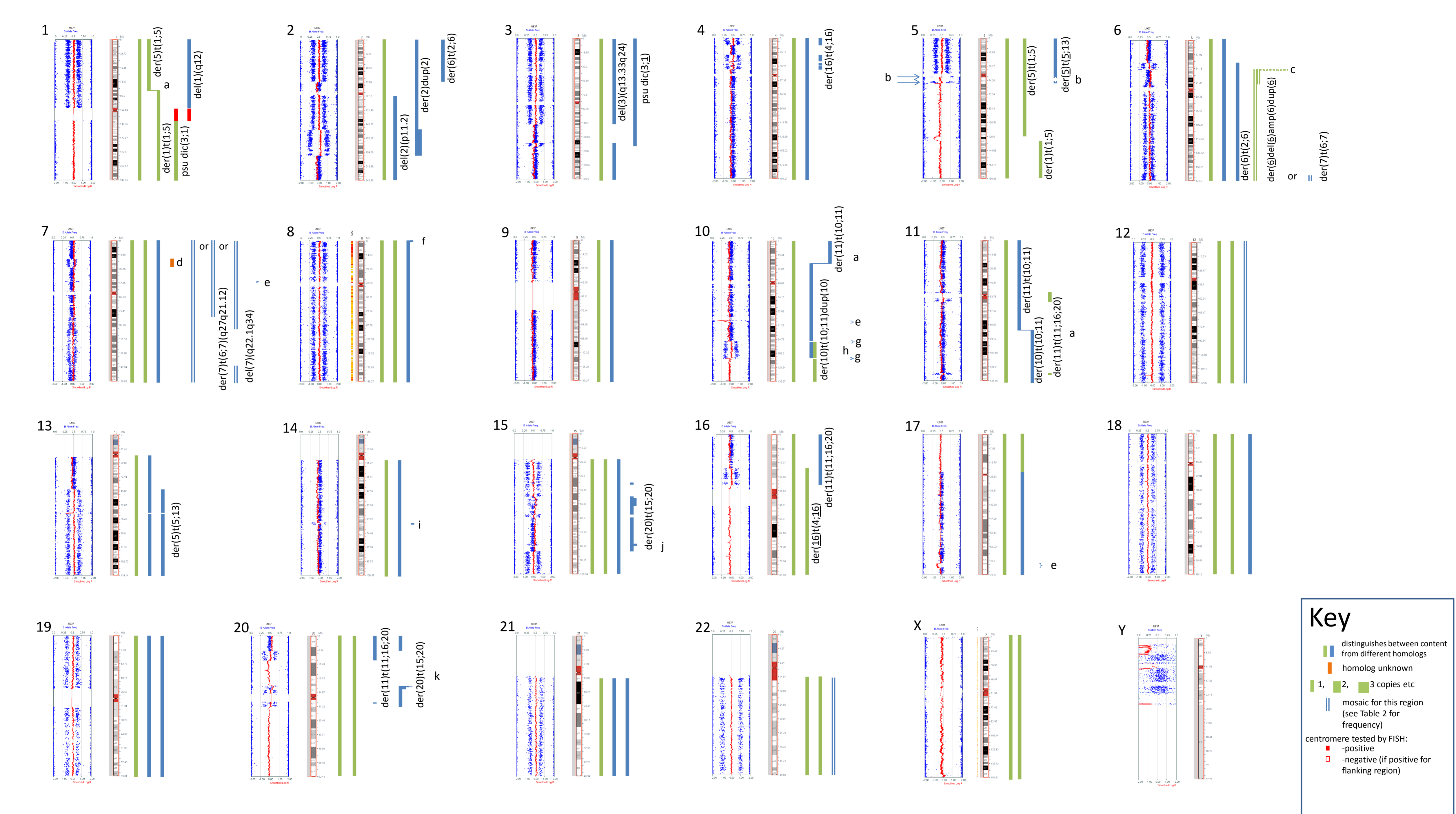

Supplement: Supplementary file 1 — Additional file 1: High resolution version of Fig. 4. [file 13039_2020_517_MOESM1_ESM.pdf]
